# Heredity

**Published:** 1913-07

**Authors:** J. W. Williams

**Affiliations:** Richmond, Va.


### HEREDITY.

By J. W. Williams, M. D., Richmond, Va.

Hereditary alcoholism is an undeniable fact.

"Alcoholism strikes a man not only in his own person, but in his own descendants."—Dr. Lunoir, Paris.

"God will visit the sins of the father upon the children unto the third and fourth generation."—Moses, Ex.: xx-5.

"Man scans with scrupulous care the pedigree of his horses, cattle and dogs before he mates them; but when he comes to his marriage, he rarely, or never, takes any such care. On the other hand, he is strongly attracted by mere wealth or rank. Yet he might by selection do something not only for the bodily constitution and frame of his offspring, but for their intellectual and moral qualities. . . . but such hopes are Utopian until the laws of inheritance are thoroughly known."—DARWIN, "DESCENT OF MAN." P. 706.

For many years it has been recognized by breeders of horses and dog fanciers that the effects of heredity are undeniable. We are indebted to Sir Francis Galton, F. R. S., for the term "Eugenics." The first Eugenic Congress has just been held in the University of London, presided over by L. Darwin, son of the great evolutionist. "It was organized to spread the knowledge of the laws of heredity—the science that deals with all the influences which improve the unborn qualities of a race with traits that are in the blood—the protoplasm." Four hundred delegates were present from all nationalities. As seed is more important than cultivation, so is the healthy hereditary germ cells (spermatozoa and ova) of more importance to the race and to society than culture and refinement. Children represent the life and vitality of their parents. All facts show that the antecedent vitality of parents and even grand-parents influences the life of a child. To be "well born" is not to be born in "high society" or to riches, or born to inherit one or all of the crowns of Europe; but at least one or two generations of healthy, normal parents must precede such birth—the ancestral germ cells must be free from contamination. The testimony of medical and sociological science corroborates the statement of the great Hebrew law-giver that the "iniquity of the fathers" affects their children, under the laws of heredity, to the "fourth generation." The father is the trustee of the germ cell, and the healthy germ cell is more important to his child than "high society" or culture and refinement. Every young man should have this fact taught him at home and in school, and it should be the first article of his social creed. If the infant at birth weighs eleven pounds, what proportion of its body-weight does its father contribute? Not one atom. Every grain of its weight came from its mother through her umbilical artery. Its father contributed its LIFE only. He is its father for this reason alone. The Creator deposited the germ cell in the father of the race and transmitted it down through the male line to his posterity. No mother can give life to her offspring. Is not the father the trustee of the germ cell (spermatozoa) in behalf of his children?

The social evil, tuberculosis, syphilis, alcohol and drug addiction all cry out in chorus for the appointment of a Chair of Eugenics in all of our universities, colleges and public schools in order that the laws of heredity may be taught the youth of our country. . . . "A universal cry of despair rises from the whole universe at the sight of diseases caused by alcohol. This invasion of alcohol ought to be regarded by everyone as a public danger, and the principle that the future of the world will be in the hands of the temperate ought to be inculcated into the masses as a truth that is incontestible."—Speech of Prof. Brouardel at Tuberculosis Congress. Every life, vegetable or animal, at its origin, passes through a germinal phase of existence and is modified, negatively or positively, by the unhealthy or healthy condition of the germ cells. The original protoplasmic cells (spermatozoa and ovum) which unite to form the beginning of the future child consist of a highly endowed protoplasm possessed of the function of "development," thereby surpassing the powers of all other cells of the human body. Now, these germ cells may be initially healthy or diseased, feeble or devitalized as it starts upon its journey of life with pure or impure blood from either one or both parents; and, in thousands of instances injured by protoplasmic poisons—syphilis, tuberculosis, and more especially by alcohol and morphine. Dr. Marion Simms declared that syphilis had "killed more of the human race than the bullet." And, 125,000 of our people in the United States alone, die in consequence of tuberculosis every year. No human being who is addicted to either of these potent drugs, alcohol or morphine, can escape the consequences of the "addiction" under the laws of heredity. We have treated fifteen physicians, among others, who were absolutely slaves to morphine and utterly unable to free themselves from its awful curse and slavery. The abnormal mental characteristics of these patients were, or seemed to be, pathognomonic and pointed unmistakably, more or less, to the pathological changes in the cells of the brain as illustrated in the microscopic photographs taken from the sensori-motor area of alcoholic patients who died at Claybury hospital; and contrasted with sections of the same area taken from normal, healthy brains of non-alcoholic patients who died at the same institution. The contrast is striking. "The nerve cells in the alcoholic brains have EXTRAORDINARILY DIMINISHED in numbers, having DEGENERATED and WASTED AWAY. The cells DEGENERATE, SHRINK and DISAPPEAR. The cells damaged in this way NEVER recover, and as far as we know are NEVER replaced."—Sir Victor Horseley's work, P. 121. This accounts for many of those ABNORMAL mental phenomena of these patients, errors of judgment, lapse of memory, hallucinations, etc. Fifty per cent. of the children of all such habitues are degenerates, as is well know; and, thus, science confirms the remarkable statement of the Mosaic Law. Sir Victor Horsley demonstrated that alcohol is found in THE FOETUS IN UTERO, and Dr. Wiglesworth declares that a "direct poisoning of the germ cell itself by the alcohol and morphine circulating in the blood, and a consequent direct injury to the cells of which this structure is composed and which by the reason of the injury are prevented from developing a stable organism. If the alcoholic poisoning of the germ cells and ovum has reached a certain degree of intensity, imbecility and even profound idiocy may be expected to result, while if of a less degree, the injury may manifest itself in the various forms of adolescent insanity, when adult life is developing, or has been attained." Dr. Potts, an eminent investigator tells us "that when a toxin finds many victims IN UTERO some of those who escape premature death must only just do so, and can hardly have an average mental and physical endowment. Some of these individuals may pass as normal up to a certain age, when, in consequence of their original poor stock of vitality, early mental or physical decay will appear." Within ten miles of my office, in Hanover county, Va., there lives a widow with three sons begotten by a drunken father, and all three perfect idiots, unable to attend even to their personal necessities. One about 40 years of age, another about 35, while the third one died. I rode out to see for myself this visitation of the sins of the father upon the first generation, under the laws of heredity. The sad, clinical picture is still upon the page of my memory—the poor, dejected mother and her helpless offspring. Other cases of like nature I could mention. Dr. Kerr states a case, typical of hundreds of others, in which was born a son, then a daughter, who were vigorous specimens of humanity, mentally and physically. After the birth of the daughter, the father became an habitual drunkard. He had four more children, of whom one was defective in mind, while the remaining three were complete idiots. In this case not only was the germ cell (spermatozoa) of the father devitalized and diseased by the alcohol circulating in his blood, but his four children yet unborn were seriously affected by the alcohol circulating in the FOETUS IN UTERO, one after another. Was not that father a criminal? Should not the State interfere here? Fourteen years ago a committee of American physicians were appointed to investigate the influence of heredity as a cause of inebriety. Over 3,000 cases were investigated. Dr. Crothers, the chairmans, says: "The heredity of inebriety is established from such studies beyond all possible question and doubt. The central conclusion, which cannot be stated too strongly, is: that the injury from alcohol to the cells and nervous tissue is transmitted to the next generation with ABSOLUTE CERTAINTY in some form or other. It may not appear always in the drink and drug symptoms, but the injury breaks out again in some neurotic trouble, defect or predisposition." Scientists properly experiment first upon the lower vertebrata, guinea pigs, rabbits and dogs chiefly, before they investigate the effects of drugs upon man. Prof. Hodge mated two alcoholized dodgs. Out of 24 puppies in four consecutive litters only four proved viable"—he then mated two normal dogs. Out of 45 puppies in eight consecutive litters 41 were viable." Prof. Latinen studied the effect of alcohol in small doses given daily for eight months to rabbits and guinea pigs. "Only 51 per cent. of the alcoholized animals lived, while in the 'control cases,' to which only water was given, 62 per cent. of the offspring lived." Dr. Feri says,

"when guinea pigs are subjected to the continuous use of alcohol during pregnancy, morbid changes are found in the brain of the offspring. There is also a marked stunting and deficiency of growth and weight. There is also a much greater tendency to disease and death."—"The Hygiene of Mind."

Prof. Domme investigated 10 alcoholic and 10 non-alcoholic families, with this result:

| | 10 alcoholic families. | 10 non-alcoholic families. |
|---|---|---|
| No. of children .... | 57 | 61 |
| Deformed ......... | 10 | 2 |
| Idiotic ........... | 6 | 0 |
| Epileptic ......... | 6 | (2 backward) |
| Non-viable ........ | 25 | 3 |
| Normal ......... | 10% | 88% |

"There are hundreds of thousands of prostitutes in this country supported at the terrible expense of the manhood of this nation. What is the price? Moral leprosy and diseased bodies! Diseased organisms manifesting themselves in their progeny, in idiocy, insanity, blindness, and other sequela. From one-third to one-half of the poverty of the United States and England results from the inefficiency produced from alcoholic indulgencies. Twenty-five to thirty per cent. of all the insane patients in the United States in our asylums owe their condition directly or indirectly to the abuse of alcohol. Dr. H. S. Williams has made a scientific investigation of statistics and concludes that, "As a criminal estimate, two-fifths of the paupers, one-fourth of the seekers of charity outside of almshouses, and nearly one-half of dependent children in America owe their deplorable condition to alcohol."—Dr. Phelps. Surely hereditary alcoholism is an undeniable fact, and "Whatsovere a man, soweth, that shall he also reap." (Gal. VI.) "Noah was a just man, PERFECT in his generations."—Gen. VI: 9. While there are some thirteen words rendered "perfect" in the Old Testament, yet the word "TAMIN" was the word selected by Moses in describing the physical perfection of the sacrificial victims in Leviticus. He used it seventeen times in this book alone. It means "WITHOUT BLEMISH." This is the word used here. (Gen. VI: 9; and Moses says, "Noah was a just man, without (physical) blemish in his generations." (BEDORTAYU, FROM DOR, A CIRCLE; the revolving years of Noah's life were "nine hundred and fifty years;" these were Noah's circles or generations). The word refers to PHYSICAL, not moral perfection. The beasts that burned and smoked upon Jewish altars had no moral character. And the high moral characteristics given Noah by Moses and St. Paul (dikaio-sune Kleronomos —"HEIR OF RIGHTEOUSNESS" (Heb. x 1:7), were all first summed up by Moses in the word "TSADDICK"—just, righteous; leaving "TAMIN" descriptive of his physical, unblemished hereditary descent, and of his uncontaminated ancestral pedigree. In other words, "Tsaddick" described his MORAL, while "TAMIN" pointed out his PHYSICAL perfection personally derived from pure Adamic ancestors. Now, spirits, beings, Elohistic, Cherubic, Seraphic or angelic have the marvelous power of materializing at will. The context shows that these spirit beings materialized as human bodies; married the "daughters of men," and their issue was a race of "Giants" of whom "Og was the remaining remnant." Deut. III: 11. The ancestral germ cells of Noah's family were of pure Adamic stock unmixed or uncontaminated with those spirit beings, who for their sin were not sentenced to "death" as the Adamic race, but to "lowest Hades," in perpetual bonds, under gloom "unto judgment of the great day," when the saints under Christ, will finally judge them ((II Pet.: II: 4 ;Cor. VI. 1-3, Jude, 6). It seems that these evil angels are not included in the Messiah's death. Heb. 11: 16—he "tasted death for every MAN."

The Episcopal Church in its Council at Lexington, Va., has just passed a resolution by a large majority, demanding a certificate from the prospective bride's family physician that the groom is free from all communicable diseases, before any clergyman can marry the parties.

This is a move in the right direction, and will, if seconded by other denominations of Christians, work out for the betterment of humanity.

Box 15, Station B.

